# Social media use informing behaviours related to physical activity, diet and quality of life during COVID-19: a mixed methods study

**DOI:** 10.1186/s12889-021-11398-0

**Published:** 2021-07-06

**Authors:** Victoria A. Goodyear, Ian Boardley, Shin-Yi Chiou, Sally A. M. Fenton, Kyriaki Makopoulou, Afroditi Stathi, Gareth A. Wallis, Jet J. C. S. Veldhuijzen van Zanten, Janice L. Thompson

**Affiliations:** School of Sport, Exercise and Rehabilitation Sciences, University of Birmingham, B15 2TT, Birmingham, UK

**Keywords:** Physical activity, Diet, Quality of life, Wellbeing, Social media, Facebook, Instagram, Twitter, Mixed methods, COVID

## Abstract

**Background:**

This mixed methods study explored how social media use informed physical activity and diet-related behaviours, and self-perceived Quality of Life (QoL) during COVID-19, and assessed the contextual factors that drive social media use for health-related behaviour change in diverse groups. During the COVID-19 lockdown periods there were reported changes to social media use and health behaviours, and this gave an opportunity to investigate potential relationships.

**Methods:**

An explanatory sequential research design of two parts was used: (1) An online survey that assessed social media use in relation to physical activity levels, diet quality and QoL (*n* = 786; *M*age 45.1 ± 19.1 (range 16–88) years; Female =69%); (2) 20 purposive focus groups (*n* = 69; *M*age = 52.88 ± 18.45 years, Female *n* = 68%) to understand the contextual factors that drive social media use for health-related behaviour change. Descriptive and thematic analysis were conducted.

**Results:**

Participants in this study reported that social media facilitated the self-management of behaviours related to physical activity, diet and QoL, through access to information to inform workouts and dietary quality, and the opportunities for interaction with peers, family members and within social groups. Contextual factors including work, home and lifestyle arrangements, pre-existing health-related knowledge and behaviours, and the perceived value of social media for health influenced the relationship between social media use and self-reported outcomes. Social media influencers, peers/family members, and official organisations influenced the application of health-related information accessed via social media.

**Conclusions:**

The evidence shows that participants were critical users of social media and were able to use social media to derive benefit for their health and wellbeing. Detailed guidance for those who use social media, as well as those who recommend and endorse social media content is required to maximise the potential of social media to support health behaviours. Future public health strategies and social media interventions should acknowledge diversity in contextual factors driving social media use for health behaviour change.

**Supplementary Information:**

The online version contains supplementary material available at 10.1186/s12889-021-11398-0.

## Background

The general public are continuously exposed to a breadth of health information through social media [1]. Over a third of world’s population use social media (38%), and usage rates are high (two-thirds or more) across many advanced, emerging and developing economies (e.g. USA, Australia, South Korea, Jordan, Tunisia) [2–4]. Through sites such as Facebook, YouTube or Instagram, a range of information related to health conditions and lifestyles is shared by the general public, professionals, influencers and/or accredited organisations [5, 6], and in areas such as physical activity and diet [1, 6, 7]. Various formats are used (memes, images, videos or text), and the interactive characteristics of social media provide opportunities to generate, comment, and/or like social media content related to health [1, 5, 8, 9], thereby permitting the creation and mobilization of diverse health information to vast audiences [6, 7]. Through social media, health information has thus become more available, shared and tailored [5], and many adults are reported to be turning to social media as a main source of health information instead of more traditional sources, such as interactions with qualified professionals in face-to-face formats [6, 10].

The increased accessibility of social media and its relevance in the lives of diverse population groups has given rise to its use in health promotion [2, 5, 8]. Leading professional health organisations, such as the World Health Organisation and Public Health England, have advocated for the use of social media to engage populations and support health-related behaviour change [11, 12]. Previous systematic reviews on social media use and/or health-related social media interventions identify that social media can positively impact physical activity and diet-related behaviours [5, 13–18]. These reviews report numerous health-related benefits of social media use for behaviour change that in addition to the accessibility of health information, include: increased interaction; peer, social and emotional support; real-time and low-cost information and interaction; and health surveillance. Overall, a key advantage of social media is that, unlike more passive forms of information, social media can help to translate knowledge into personalised action at specific points in time, when it becomes ‘needed’ and without the need for a ‘physical’ instructor or ‘physical’ health setting [9, 19].

Despite evidence of the value of social media in health promotion, social media use has the potential to negatively impact on health-related behaviours [5, 8–10, 18]. There is abundant literature on the negative impacts of social media use in areas such as: reduced physical and psychological health due to sedentary lifestyle, loss of sleep, poor dietary habits and cognitive impairment [8, 9]; risks for mental health, such as anxiety, depression, stress, low mood and body dissatisfaction [18, 20, 21]; and impact on cognitions, such as negative self-perception and social isolation [8, 9, 20]. It has been reported that social media use has the potential to lead to harm due to: excessive time spent on social media leading to the displacement of other health and wellbeing promoting activities; the quality and reliability of public health information; inaccurate interpretations of information; the communication of incorrect advice; and privacy, confidentiality and data security [5, 8, 18, 20]. Hence, many professionals recommend approaching social media for health promotion with caution [5].

While studies report on the benefits and risks of social media for health promotion, there is no robust evidence on the key characteristics of social media use that are associated with positive physical activity and diet-related behaviours, and the contextual factors that drive social media use for health-related behaviour change [9, 13, 16]. Most evidence on social media use and/or interventions has been generated from at risk and/or clinical populations groups, and there is little understanding of how non-clinical groups, such as those physically active, may benefit from social media [5, 8]. Evidence also tends to be dominated by research on Facebook and/or passive forms of communication that are defined by the intervention team [13, 15, 16], where the use of contemporary mediums (e.g. SnapChat, Instagram, and WhatsApp) is limited. In turn, our understanding of how to design interventions to reach mass audiences, and how to tailor social media interventions to specific groups and subgroups is currently insufficient.

During the COVID-19 lockdown periods of 2020 there were reported changes to social media use and physical activity and diet behaviours across international contexts and population groups, and this provided an opportunity to investigate possible relationships. COVID-19 altered many individuals’ typical lifestyle behaviours, such as, restricted movement, altered accessed to certain food types, enforced closure to exercise and sport facilities [22–25], home working, home schooling and increased leisure time in the home [23, 26–29]. These lifestyle changes may have led to changes in health behaviours and the use of social media. Furthermore, a plethora of freely accessible health information (such as workouts and recipes) was shared through social media during COVID-19 lockdown periods, and this had the potential to improve individuals’ self-perceptions of their Quality of Life (QoL) through supporting their engagement with typical lifestyle behaviours, albeit online [30, 31]. Evidence generated during COVID-19 lockdown periods of 2020 has reported on a general tendency for technology and social media use to increase [26–28], and overall physical activity levels and high quality nutritional intake to decline [23, 29, 32, 33]. However, the evidence is not definitive, and there is some evidence to suggest that physical activity and diet-related health behaviours improved [25, 32]. The contextual factors that influence social media use, physical activity and diet-related behaviours for diverse groups are also unclear in current studies, and there is limited evidence on the relationships between social media use and health-related behaviour change related to physical activity, diet and quality of life [25, 29, 31, 32].

### Aims and research questions

The aim of this study was to explore how social media use informs physical activity and diet-related behaviours, and self-perceived QoL [34], and to assess the contextual factors that drive social media use for health-related behaviour change for diverse groups. The research questions guiding this study were:

During the COVID-19 lockdown period (March 2020–June 2020):

1. What were the self-reported changes in physical activity levels, diet quality, and QoL?
2. What health information was accessed by participants through social media and used to inform behaviours related to physical activity, diet, and QoL?
3. How do participants with different living and working situations (self-isolating, working from home/leaving home to work) and/or physical activity levels (low, high) differ in their uses of social media to access health information?

## Materials and methods

The study adopted a mixed methods, explanatory sequential research design [35]. The mixed methods approach aligned with the research questions and provided a means to address the identified gaps in the literature by measuring self-reported changes in social media use and health behaviours (quantitative) and by contextualising how and why social media use, physical activity, diet and quality of life were altered during the COVID-19 lockdown period (qualitative). Quantitative data were collected and analysed from an online survey, and the findings were used to inform qualitative data collection using focus groups.

### Expert stakeholder consultations

Seven focus group (FG) consultations were conducted with 50 stakeholders (researchers, professionals/practitioners in sport, exercise, public health and education) who represented people from diverse age and activity groups (e.g. young adults, older adults, active/inactive) and specific health condition groups (e.g. diabetes, rheumatoid arthritis). Engaging a range of stakeholders and capturing their diverse experiences and viewpoints enhanced the accountability and transparency of the research as well as increased its relevance for the intended audiences [36]. Additionally, the FGs helped to determine appropriate recruitment strategies, methods and measures. Face validity and content validity of the survey were determined through a pilot phase with 17 participants (age range 30–72 years, 100% White, gender not asked) in the UK who completed the survey (*n* = 12) and/or participated in a FG interview (*n* = 5). The pilot phase informed changes to survey questions about social media use and the topic guide for FGs.

### Recruitment

Convenience sampling was used to recruit participants to the online survey from a range of diverse demographics, in the UK and internationally (including age, sex, gender, ethnicity, working status, and level of deprivation for participants living in England and Wales as represented using the Index of Multiple Deprivation^1^ [IMD]). Recruitment primarily occurred through: (i) advertisements on social media, and via an online video with embedded hyperlink to the survey; (ii) through national and international organisations and agencies with whom the research team had established working relationships; and (iii) through individuals who participated in the expert stakeholder consultations. Participants who completed the survey were purposively invited to participate in FGs (details provided in the FGs sub-section).

### Ethics

Ethical approval was granted from the University STEM ethics committee [ERN_ 20-0616A] and all methods were conducted with the provisions within the ethical approval. Participants were aged 16+ years, and all provided written informed consent digitally. All participant data was anonymised during the reporting of research findings.

### Data collection

#### Online survey

The online survey was constructed and administered using the 2021 Jisc Online Survey platform. Apart from demographic questions (e.g. age, gender, ethnicity) and questions related to their living situation (see Table 1), this survey included questions related to social media use and health behaviours. Data were collected at one time point and aimed to measure participants’ self-reported changes in physical activity and diet-related behaviours, self-perceived QoL and social media use during the COVID-19 lockdown period. Bespoke questions were created for physical activity, diet, and self-perceived QoL due to the study aim of assessing the extent of self-reported changes in these behaviours specifically during the COVID-19 lockdown period. Previously used surveys on social media use were employed to understand engagement with social media in terms of time, mediums, and content (35-item questionnaire, [9]).

**Table 1:** Descriptions of population groups based on different experiences of COVID-19 social distancing measures

Category	Explanation
Self-Isolating (SI)	Self-isolation is when you do not leave your home. There are a few reasons people self-isolate, and these include: (i) due to medical advice; (ii) due to the development of symptoms related to COVID-19; or (iii) due to having been in contact with people who have developed symptoms related to COVID-19.
Leaving Home to Work (W)	Refers to individuals who leave their house to go to work, to get medication, to do food shopping and daily exercise or leaving the house as usual.
Working or Studying from Home (H)	Refers to individuals who self-identified themselves as only leaving their house to get medication, to do food shopping, and do daily exercise.

The categories were developed based guidance issued by the UK on social distancing [37, 38] and evidence on individuals behaviours during COVID-19 lockdown periods [23]

Sixty-one questions were included. Participants were asked to indicate the amount of time they had spent per day on social media (< 1 h, 1-2 h, 2–4 h, 4–5 h, > 5 h), as well as the social media platforms they used (e.g., Facebook, Twitter, WhatsApp). Participants were also asked to indicate to what extent they agreed that social media was a good source of information on physical activity, diet, and QoL during the COVID-19 lockdown, and whether they thought social media during the lockdown had a positive influence on physical activity, diet quality, and QoL. All these questions were scored on a 5-point Likert scale, ranging from 1 – strongly disagree, to 5 – strongly agree. Changes in social media use, physical activity and QoL were assessed on a 5-point Likert scale, ranging from 1 – decreased a lot to 5 – increased a lot. Change in overall quality of diet and eating behaviours were scored on a 5-point Likert scale, ranging from 1 – strongly disagree to 5 – strongly agree. Participants were also asked if they had seen, read, or watched social media posts related to physical activity, diet, and QoL, and if so, if they had acted on the information in that post. Finally, participants were asked to provide an example of a social media post that had been particularly influential on their attitudes, knowledge and behaviours. The survey can be accessed from Supplementary File A (the survey). The survey was administered during the initial lockdown period in the UK (March–June 2020) between 26th April 2020 and 15th June 2020.

#### Survey analysis

Frequency analyses were completed for all variables related to changes in behaviour, as well as views and experience of social media use (social media, physical activity, diet/nutrition) for the whole sample and the separate COVID categories. Content analysis was used to analyse free text responses of social media posts and informed by established methods for understanding content, communication, and influence of social media posts [39]. Data were firstly coded in relation to health behaviour category (physical activity, diet and QoL). The frequency of social media posts was then identified in relation to source (i.e., celebrity/influencer or national and local organisations), and the social media accounts referred to the most within each category were selected and analysed in relation to profile, qualifications, location, and social media platforms.

#### Focus groups

The aim of the FGs was to explore self-reported changes in physical activity levels, diet quality and QoL in relation to social media use, and the contextual factors that drive social media use for health behaviour change. Following the explanatory sequential design [35], and informed by literature [23, 24], our expert stakeholder consultations, and the survey data, a purposive sample of participants were selected and organised into 8 different groups that represented: (i) no changes in dietary quality, (ii) decreases in physical activity levels; (iii) increases in physical activity levels; (iv) low physical activity levels prior to the lockdown period; (v) high physical activity levels prior to the lockdown period; (vi) use of social media as a positive resource for physical activity, diet and/or QoL; (vii) increase in time spent on social media during the lockdown period; and (viii) self-isolating individuals. One hundred and three participants were invited to participate in the FG via email. In total 20 focus groups with 69 participants were completed (*M*age = 52.88 ± 18.45 years, Female *n* = 68%; 12 = self-isolating, 9 = leaving home to work, 48 = working/studying from home). Health behaviours and COVID categories were further understood from FG transcripts to ensure coherence with survey sampling and as a measure of quality [40]. Overall, the sample of 20 FGs was broadly representative of the main survey population (Table 2). A description of the 8 FG categories and participant characteristics in each group are reported in Supplementary File B.

**Table 2:** Participant characteristics and social media use for the whole sample and split by COVID category

	All	SI	W	H
n	786	62	190	527
Percentage of sample		8	24	67
Mean (SD) Age (years)	45 (19)	58 (21)	41 (15)	45 (20)
Gender – n (%)				
Female	531 (68)	42 (68)	114 (60)	373 (71)
Male	235 (30)	16 (26)	71 (37)	144 (27)
other	3 (0.3)	0	0	3 (1)
Unknown	17 (2)	4 (6)	4 (2)	7 (1)
Ethnicity – n (%)				
White British	592 (75)	52 (84)	132 (70)	407 (77)
Other White	104 (13)	7 (11)	36 (19)	58 (11)
Other	75 (10)	1 (2)	18 (9)	54 (10)
Unknown	15 (2)	2 (3)	4 (2)	8 (2)
Country – n (%)				
UK	597 (76)	52 (84)	119 (63)	425 (81)
Other	98 (12)	3 (5)	39 (21)	53 (10)
Unknown	91 (12)	7 (11)	32 (17)	49 (9)
Mean (SD) IMD (UK only)	6.3 (2.6)	6.1 (2.8)	64 (2.7)	6.3 (2.5)
**Time on Social Media**				
Decrease	62 (8)	4 (7)	12 (6)	45 (9)
No Change	157 (20)	5 (8)	34 (18)	117 (22)
Increase	563 (72)	53 (86)	141 (74)	364 (69)
Missing	4 (1)		3 (2)	1 (0.2)
**During Lockdown**				
< 1 h	111 (14)	13 (21)	16 (8)	82 (16)
1–2 h	233 (30)	20 (32)	65 (34)	148 (28)
2–4 h	209 (27)	14 (23)	55 (29)	137 (26)
4–5 h	105 (13)	7 (11)	26 (14)	70 (13)
≥ 5 h	113 (14)	7 (11)	22 (12)	83 (16)
Missing	15 (2)	1 (2)	6 (3)	7 (1)
**Social Media Site**^**a**^				
Facebook	548 (70)	39 (62)	143 (75)	361 (69)
Twitter	278 (35)	11 (18)	71 (37)	195 (37)
YouTube	422 (54)	33 (53)	102 (54)	282 (54)
WhatsApp	590 (75)	45 (73)	138 (73)	401 (76)
Instagram	379 (48)	17 (27)	106 (56)	252 (48)

Note: ^a^ – participants could select more than 1 option; Self Isolating (SI), Leaving home to work (W), Working or Studying from Home (H); Index of Multiple Deprivation (IMD); United Kingdom (UK)

A consistent format was used across the 20 FGs that included semi-structured interview and elicitation techniques. There were 8 questions focused on: (i) social media engagement; and (ii) health behaviours (physical activity, diet/nutrition) and QoL. Descriptive data and images of social media posts reported by participants in the online survey were used as prompts. The FGs were led by a trained researcher and took place online using Zoom video conferencing (duration = 42–65 min). MP3 files were downloaded and later transcribed verbatim.

The FG recordings were transcribed and coded using NVivo9 software. Analysis was inductive and followed a 6-step thematic data analysis approach: (i) familiarisation; (ii) coding; (iii) theme searching; (iv) reviewing themes; (v) defining and narrowing themes; and (vi) reporting/explanation [41]. Thematic maps were produced to illustrate the findings. Qualitative analysis was led by author VG. The process of reviewing, narrowing and reporting/explaining themes was completed by interdisciplinary authors AS and KM. This deliberative approach incorporating multidisciplinary views and perspectives of authors from different disciplinary backgrounds served as a marker of quality and rigour in the construction of themes [40, 42].

## Results

A contiguous approach was followed to interpret and report findings from the mixed methods data set [35]. Accordingly, we report quantitative and qualitative data separately.

### Participant characteristics and social media use

Seven hundred and eighty-six participants completed the survey, with most participants from the UK (76%) (Table 2). The *M*age of the sample was 45.1 ± 19.1 (range 16–88) years, with much of the sample female (F = 69%), and of White British ethnicity (77%), with 13% from other White backgrounds, and 10% from Black, Asian, and Minority Ethnic groups. The mean IMD score for participants from the UK (score of 1 indicating the 10% most deprived areas, and a score of 10 indicating the 10% least deprived areas) was 6.3 ± 2.6. Most of the participants in the sample reported working/studying from home (67%), with some identified as leaving the home for work (24%) and fewer self-isolating (8%) (Table 2) – and these are broadly comparable to the population demographics in the UK during the COVID-19 lockdown period [37, 38]. The demographics of the different groups were similar, except for age, with participants in the self-isolation group being older (see Table 2).

**Fig. 1:**
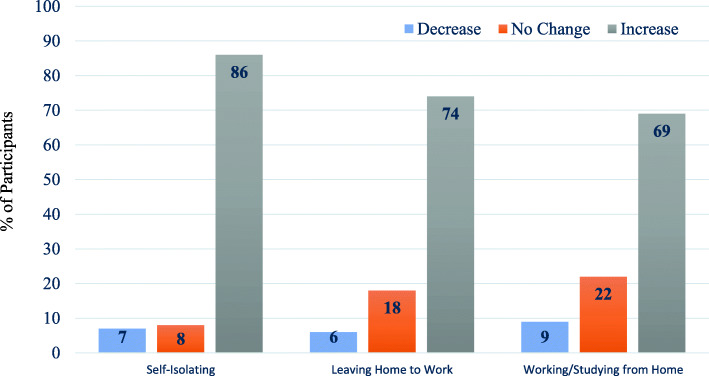
Percentage of participants within each social distancing category reporting a decrease, no change, or an increase in social media use during the COVID-19 lockdown period

The time spent on social media and the main social media sites used are reported in Table 2. Across the survey sample, time spent on social media predominantly increased during lockdown (72%). Most participants tended to spend between 1-2h (30%) and 2–4 h (27%) on social media, and the most popular media were WhatsApp (75%), Facebook (70%) and YouTube (54%). A greater proportion of individuals who were self-isolating reported an increase in time spent on social media during the COVID-19 lockdown period in comparison to groups who were leaving home to work or working/studying from home (Fig. 1).

### Self-reported changes in physical Activity levels, diet quality and quality of life

Table 3 reports the changes in physical activity, diet quality, and QoL for all participants, as well as split by COVID category. Approximately half of all participants reported a decrease in physical activity and QoL, with 56 and 65% of people in the self-isolation group reporting a decrease in physical activity and QoL, respectively. Approximately 35% of participants reported an increase in physical activity and about 20% an increase in QoL. Finally, 44% of participants reported no change in diet quality, with about a third of participants reporting an improvement in their diet quality during lockdown.

**Table 3:** Changes in physical activity, diet quality, and quality of life during lockdown for the whole sample and split by COVID category

	All	SI	W	H
	*n* = 786	*n* = 62	*n* = 190	*n* = 527
**Physical activity**				
Decreased a lot	166 (21)	18 (29)	34 (18)	112 (21)
Decreased a little	211 (27)	17 (27)	50 (26)	141 (27)
No Change	125 (16)	7 (11)	35 (18)	82 (16)
Increased a little	182 (23)	15 (24)	48 (25)	119 (23)
Increased a lot	96 (12)	5 (8)	20 (11)	71 (14)
Missing	6 (1)		3 (2)	2 (0.4)
**Diet quality**				
Strongly disagree	23 (3)	1 (2)	6 (3)	16 (3)
Disagree	156 (20)	15 (24)	40 (21)	98 (19)
Neutral	345 (44)	23 (37)	84 (44)	237 (45)
Agree	219 (28)	17 (27)	54 (28)	147 (28)
Strongly agree	39 (5)	6 (10)	6 (3)	26 (5)
Missing	4 (1)			3 (1)
**Quality of Life**				
Decreased a lot	76 (10)	10 (16)	14 (7)	52 (10)
Decreased a little	343 (44)	30 (48)	78 (41)	232 (44)
No Change	202 (26)	12 (19)	58 (31)	130 (25)
Increased a little	127 (16)	10 (16)	32 (17)	84 (16)
Increased a lot	33 (4)	0	7 (4)	26 (5)
Missing	5 (1)		1 (1)	3 (1)

Note: all data presented as number (%); Self Isolating (SI), Leaving home to work (W), Working or Studying from Home (H)

### Types of health-related information accessed through social media and used to inform behaviours related to physical activity, diet quality, and QoL

**Fig. 2:**
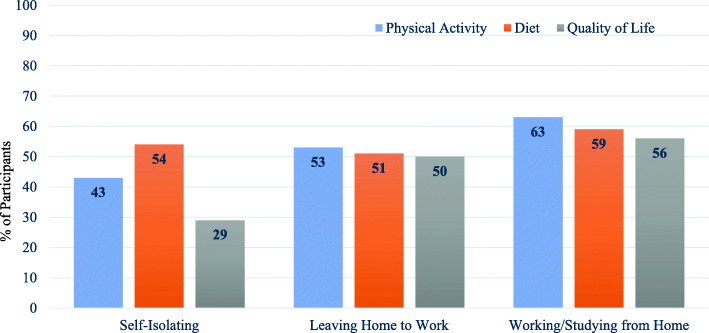
Percentage of participants within each social distancing category reporting acting on a social media post related to physical activity, diet, or quality of life

Table 4 reports perceptions of social media and use of social media for physical activity, diet and QoL. The overall perception about social media being a good source of health-related information varied, with participants tending to agree and/or have neutral views. The participants tended to have more neutral views about whether social media was a good source of information for diet and QoL, whereas more participants tended to agree that social media was a good source of information for physical activity. A greater proportion of the participants reported seeing social media content that related to physical activity (65%) than diet (53%) or QoL (30%). Yet while physical activity content was reported as being seen the most, a greater proportion of the participants reported acting on social media content (i.e., using the information to inform their behaviours) that related to diet (56%) and QoL (53%) compared to physical activity (41%). A higher percentage of individuals working or studying from home reported acting on social media content related to physical activity, diet and QoL as compared to the self-isolating group and/or those who reported leaving home to work (Fig. 2).

**Table 4:** Perceptions of social media and use of social media for physical activity, diet, and quality of life during lockdown for the whole sample and split by COVID category

	All	SI	W	H
	*n* = 786	*n* = 62	*n* = 190	*n* = 527
**SM good source**				
Strongly Disagree	50 (6)	7 (11)	13 (7)	30 (6)
Disagree	95 (12)	2 (3.2)	29 (15)	63 (12)
Neutral	303 (39)	32 (52)	61 (32)	207 (39)
Agree	267 (34)	16 (26)	67 (35)	183 (35)
Strongly agree	65 (8)	3 (5)	20 (11)	41 (8)
Missing	6 (1)	2 (3)		3 (1)
**SM good source for PA**				
Strongly Disagree	51 (7)	4 (7)	11 (6)	36 (7)
Disagree	101 (13)	10 (16)	23 (12)	67 (13)
Neutral	303 (39)	21 (34)	71 (37)	195 (37)
Agree	260 (33)	19 (31)	67 (35)	172 (33)
Strongly agree	77 (10)	5 (8)	18 (10)	54 (10)
Missing	8 (1)	3 (5)		3 (1)
**See SM post on PA**				
No	275 (35)	32 (52)	60 (32)	181 (34)
Yes	511 (65)	30 (48)	130 (68)	346 (66)
Acted on post	302 (41)	14 (43)	69 (53)	219 (63)
**SM good source for diet**				
Strongly Disagree	48 (6)	4 (7)	10 (5)	34 (7)
Disagree	133 (17)	15 (24)	27 (14)	91 (17)
Neutral	384 (49)	26 (42)	98 (52)	257 (49)
Agree	193 (23)	12 (19)	49 (26)	121 (23)
Strongly agree	26 (3)	1 (2)	6 (3)	18 (3)
Missing	12 (2)	4 (6)		6 (1)
**See SM post on diet**				
No	366 (47)	36 (58)	84 (44)	246 (47)
Yes	416 (53)	26 (42)	106 (56)	280 (53)
Acted on post	233 (56)	14 (54)	54 (51)	164 (59)
**SM good source for QoL**				
Strongly Disagree	60 (8)	2 (3)	18 (10)	40 (8)
Disagree	141 (18)	11 (18)	29 (15)	101 (19)
Neutral	388 (49)	29 (47)	97 (51)	259 (49)
Agree	165 (21)	15 (24)	41 (22)	108 (21)
Strongly agree	17 (2)	1 (2)	2 (1)	13 (3)
Missing	15 (2)	4 (6)	3 (2)	6 (1)
**See SM post on QoL**				
No	552 (70)	48 (77)	130 (68)	367 (70)
Yes	234 (30)	14 (23)	60 (32)	160 (30)
Acted on post	124 (53)	4 (29)	30 (50)	90 (56)

Note: all data presented as number (%); *SI* Self Isolating, *W* Leaving home to work, *H* Working or Studying from Home, *SM* Social Media, *PA* Physical Activity, *QoL* Quality of Life

In total, 358 social media posts were identified from the survey that participants reported using to inform their behaviours related to physical activity, diet, and/or QoL (see Table 5). Of the examples shared, 160 were related to exercise (e.g., online workouts [High Intensity Interval Training [HIIT], yoga, weights/resistance, running challenges), 37 were related to diet (e.g., recipes, advice for specific diet patterns or types [vegan, vegetarian, high Vitamin D, anti-stress] and supplements) and 96 were related to QoL (e.g., hobbies, clubs, social interaction, meditation). The remainder focused on generic social media use, such as mediums or platforms (e.g. Facebook Live). Of the 358 posts, 115 related to specific social media accounts or individuals. Of those 115 posts, celebrities/influencers and personal trainers/fitness coaches were mentioned the most (*n* = 73), followed by national health/sport organisations (*n* = 12). To a lesser extent, celebrity doctors (*n* = 5), celebrity chefs (*n* = 3) and posts by local health/sport organisations (*n* = 7, e.g., local gyms, sports clubs) were also mentioned. Fifteen of the 115 posts were classified as ‘Other’ and included blogs, medical advice, and news articles about topics such as Cannabidiol oil. The account referred to most frequently across all categories was reported in relation to physical activity, and is typically identified as a celebrity/influencer personal trainer.

**Table 5:** Social Media Content Reported to be Used

Category	Example Descriptions of Accounts on Instagram, Facebook, YouTube^a^
Celebrity/Influencer Personal Trainer/Fitness Coach (*n* = 73, 63.5%)	On a mission to make the world fitter, healthier and happier. I post weekly home workouts to help you get stronger, healthier and happier
	Daily Live Workouts, Qualified Personal Trainer 3 x Bestselling Author, Women’s Health Columnist, Women’s Aid Ambassador, My Podcast – Here to lift you up
	If you want to look like an athlete you’ve got to train like an athlete!. This is where you can find all the latest FREE workouts, nutrition and training advice on your way to a healthy, leaner and more muscular athletic body by training like an athlete. Learn from the physical therapist and strength training coach and exactly what he does with his professional athlete and celebrity clients. Put the science back in strength to build muscle, keep your muscles and joints healthy and to improve your overall athleticism.
	I make people sweat. Subscribe to my channel and find weekly workout videos, healthy food recipes and other fun videos! I’ve got FREE workout programs on my channel with workout calendar schedules so check it out!
	Get trained by me, Fitness is my number one love, but on this channel you will also find all things beauty, skincare, food & fashion, as well as workouts, training tips, healthy recipes and MORE.
	Our mission is to connect as many people as possible through high-quality free yoga videos. We welcome all levels, all bodies, all genders, all souls! Browse our library of free yoga videos to find a practice that suits your mood or start a journey toward healing. If you’re brand-new to yoga, check out my Yoga for Beginners and Foundations of Yoga series. These are designed to give you the tools to build a happy, healthy at home yoga practice. If you’re ready to work up a sweat, try the Yoga for Weight Loss or Total Body Yoga playlists. Calm and relieve a tired mind and body. Create space. Tone and trim. Cultivate self-love. Contemplate. Reflect. Make time for you. Go deeper, have fun. Connect. Fall off the horse and then get back on. Reconnect. Do your best, be authentic and FIND WHAT FEELS GOOD. I got your back and this community rocks. Jump on in! You don’t even have to leave your house.
Celebrity Doctor (*n* = 5, 4.3%)	Keeping Health simple. Podcast: Feel better, live more (25 million listeners). Author: 4 x Sunday times Bestsellers, Radio Presenter
Celebrity Chef (*n* = 3, 2.6%)	Chef and Dad. Keep Cooking Daily – Weekdays from mid-day over on Facebook live.
National Health/Sport Organisation (*n* = 12, 10.4%)	To help adults live more healthily. It is a campaign that aims to encourage adults across the country avoid future diseases caused by unhealthy lifestyles. It will help people move more, eat well, drink less alcohol and be smoke free. It provides tools, support, and encouragement every step of the way, to help improve your health right away. Search online to visit our site and take the ‘How Are You’ online health quiz. You can find free support and advice to help you look after yourself.
Local Health/Sport Organisation (*n* = 7, 6.1%)	An iconic new club providing world-class opportunities for everyone in the region, from beginner to elite athlete.

^a^The example descriptions of accounts reported here, are all of those reported more than once from the online survey by participants in a free-text response to health-related social media posts that they deemed to be influential to them. Description has been taken from account bio information and associated links on Instagram, Facebook and/or YouTube; descriptions have been edited in some instances to spell out acronyms and to anonymise the account

### Contextual factors influencing social media use for health-related behaviour change

Thematic analysis of the FGs identified 5 themes highlighting the contextual factors that influenced social media use during the COVID-19 lockdown period: free access/easy to engage, work-home-health balance, creating e-local communities, authentic experiences, and recommended content. The themes are reported below with illustrative quotes (see also Supplementary File B).

#### Free access/easy to engage

Economic instability and the perceived opportunities of social media to be used for information and interaction, but unfamiliarity with social media (i.e. limited or no prior use) were barriers to the use of social media that were overcome by: (a) the provision of free social media accounts (e.g. Facebook, Zoom) and health-related content that was accessible across devices (Tablet, phone) and platforms (e.g. Android, Apple); and (b) content delivered in a video format that was short in duration, requiring no equipment or minimal/inexpensive recipe ingredients.

*YouTube, it’s just free, you can just access it whenever you want, there’s no cost to it …*. *That’s another thing as well, because I’m not going to the gym, and I’ve frozen my membership because you’ve got to save money where you can … So, I think that’s a big thing about it is the flexibility of it, and the fact that there’s no cost to it either. (Female, 35–44 years, Social Media as Positive Resource Interview Group)*

#### Work-home-health balance

Less time spent commuting/travelling to work and more leisure time, combined with the ease of completing online/social media workouts at home and with the family, were key drivers for using social media to access and act on physical activity and diet information.

*You haven’t got the whole commute, you haven’t got the rush of, “Oh, god, I’m so hungry”. When I get in from work I want to cook dinner and go to bed, you haven’t got that. Our whole routines have been flipped, so everybody’s got more time on their hands. Also, I think, people are maybe realising, “Oh, actually, I don’t have to go to the gym to work out” or “I don’t have to join a class to work out.” … We both [referring to partner] do ‘it’ [specified social media workout] at 9:00 every morning, and we’ll do it together. (Female 16–24 years, Physical Activity Increase Interview Group)*

#### Creating E-local communities

Access to community-based health-related contexts (e.g. sport and exercise facilities, cafes/restaurants, nutrition clubs, scouts and guides) were restricted during the lockdown, but local organisations and groups of individuals used social media to run their typical exercise and cooking sessions online (e.g. Zoom cooking parties, Facebook Live Pilates) and, in turn, these provided a sense of community, a ritual/schedule, and enabled participants to maintain social connections, influencing overall QoL.

*We’ve got a WhatsApp running group, and I’ve got a Facebook Messenger running group. We all kept putting ‘I’ve done this, I’ve done that’, not the same as running with your friends or doing the parkrun [free, weekly community running events], but it helps (Male, 45–54 years, Diet Change Interview Group)*

#### Authentic experiences

The perceived need to improve health-related behaviours coupled with restricted social interactions outside of households (e.g., family and friends) were drivers for the use of social media. Live physical activity and diet videos shared on social media were particularly helpful resources to inform physical activity and diet behaviours, when presenters (e.g., celebrities/influencers) portrayed their real lives, personalities, and everyday bodies (rather than unattainable elite) by being warm, calm, and laidback and by opening their doors to their homes (e.g., babies and dogs), and in turn, this promoted feelings of ‘in this together’ and reduced feelings of isolation.

*Has to be his personality [referring to a celebrity influencer/personal trainer] … I really liked it when he’d injured his wrist, the fact he kept going … and I liked it when he involved his wife in it … I just admire him for that. I just think he’s a nice, warm, likeable person, really. Like a friend that you want to try and please. (Female 65–74 years, Social Media as a Positive Resource Interview Group)*

#### Recommended content

Misinformation (including conflicting health advice from media, government, and potential conspiracy theory sources) and diverse health conditions, health-related knowledge and experience with social media impacted on the willingness and interest to engage with and use health-related social media content. However, recommendations or endorsements by peer/family members and/or official organisations that participants were affiliated with and/or trusted (e.g., National Health Service (NHS), Diabetes UK, Runners World, university-related accounts) influenced participants to use and engage with content related to physical activity, diet and/or QoL.

*There’s a specific guy who I use all the time who a friend put me onto, who’s very, very good. He lays it all out with science. I think the guy’s got a biology degree, and he lays it all out in a way that you can actually understand. Rather than just being like, “Oh, this is the movement you need to do”, he actually lays out why you need to do it. (Male, 16–24 years, Social Media Increase Interview Group)*

### Alternative uses of social media related to physical activity levels and social isolation status

Participants reported a diverse experience of the impact of the five contextual factors on how they used social media related to physical activity levels prior to the lockdown and self-isolation status during the lockdown. A description of the FG participants and their physical activity levels and self-isolation status are reported in Supplementary File B (Table S3). Thematic maps to illustrate these findings are reported in Supplementary File C.

#### Self-isolating

These participants reported contextual challenges related to restricted access to physical activity spaces, supermarkets and social interaction outside of households. Social media helped adults who were self-isolating to address these challenges by providing a medium to engage with online workouts to maintain physical activity behaviours, and by providing a space to interact with family members and peers to support their overall QoL. Social media was not a resource for all adults who were self-isolating, and was not used to inform diet quality. Furthermore, these adults were sceptical of using social media in relation to the relevance of content to senior bodies, whether information was recommended by peers and/or family members, and whether issues related to privacy and misinformation were appropriately addressed.

#### High physical activity levels

Adults who had high physically activity levels prior to the lockdown experienced contextual challenges related to restricted access to local community and/or professional sport or physical activity contexts, that resulted in limited social interaction (e.g. spectator, teams) and access to physical spaces (e.g. closure to gyms, access to professionals). Social media was primarily used as a substitute/add-on for social contact related to physical activity/sport (i.e. groups, networks, teams and clubs), through WhatsApp groups and/or apps and challenges designed for running or cycling (e.g. Strava). These adults rarely turned to social media for health-related information to inform their physical activity and diet behaviours, as they perceived they had a high level of knowledge about how to maintain their behaviours. For some of these adults, social media was used to purchase gym equipment and/or engage with professionals that they trained with prior to the lockdown.

#### Low physical activity levels

For adults who had low physical activity levels prior to the lockdown, COVID-19 re-emphasised the importance of physical and mental health, and these adults were motivated to improve their lifestyle behaviours related to physical activity and diet. Social media helped these adults by providing a medium to engage with online workouts with individuals they perceived to be engaging and supportive (e.g. celebrity personal trainers). Social media was also used to access recipes and to engage with community-based online cooking classes to improve their diets and experiences of QoL. The use of social media and changes to physical activity and diet behaviours during the COVID-19 lockdown period were re-enforced due to the accessibility of social media and its contents, and changes to these adults work-home-lifestyles, including working from home, less time spent commuting, and increased leisure time with the family.

## Discussion

Participants in this study reported that social media facilitated the self-management of behaviours related to physical activity, diet and QoL, through access to information about workouts and dietary quality, and opportunities for interaction with peers, family members and within social groups. The relationship between social media use and self-reported outcomes associated with physical activity, diet, and QoL varied and was influenced by contextual factors, including work home and lifestyle arrangements, pre-existing health-related knowledge and behaviours and the perceived value of social media for health and wellbeing. The findings reveal that: (a) people respond to similar forms of social media and health-related content in different ways; and (b) there are contradictions in the ways in which people attach value to social media and are influenced to use social media content or not. Hence, this study provides new evidence to inform how social media can be used to reach and engage diverse population groups to support health-related behaviour change. Overall, this study illustrates the importance of mixed methods research to understand the reasons and context for social media use in order to explain relationships between social media and health-related behaviours.

In relation to the characteristics of social media use that inform behaviour change, this study adds new understandings on the value of using social media for information and interaction [5, 13–16, 18, 31]. New evidence is provided on: (i) the format, content, and source of health-related social media information that participants engage with and use; and (ii) the perceived value of the immediacy of interaction in relation to social dimensions associated with QoL. The participants engaged with videos of short duration, that were focused on physical activity and that were predominantly shared by social media influencers. Furthermore, the data reported on how participant engagement with live (synchronous) videos and stories led to the maintenance and development of communities and social connections within organised clubs or groups, that, in turn, reduced experiences of isolation and supported social relationships. Accordingly, it was evident that, contrary to popular opinion [18], many participants are critically aware users of social media, who are able to navigate a plethora of social media content to derive benefit for their health and wellbeing, and in the areas related to physical activity, diet and QoL.

The findings illustrated that a key driver of engaging with and using health-related information accessed from social media was social influence from others. Social influence was observed from three main sources: (i) social media influencers; (ii) peers/family members; and (iii) official organisations. Like previous research [39, 43], social media influencers gained the trust and friendship of their followers by positioning themselves as experts, sharing expert information, showcasing personal experience and by creating a sense of perceived relatability, intimacy, familiarity, and sympathy. Peers and family members and official organisations operated through informational social influence [44], such that participants were more likely to use health-related information accessed from social media if their peers or family members used it, and/or if an official organisation endorsed the content. Together these three social influences were powerful agents of behaviour change, particularly at a time point in need, and during the COVID-19 pandemic when social influence was reported to heighten adherence to public health advice [45].

While many of the social influences were likely to have had a positive effect on physical activity, diet, and QoL, there was also the potential for physical and mental harm. Previous research has shown that, for some people, the use of social media content related to workouts, exercises or diets accessed from influencers and/or via peers and family members can lead to musculoskeletal injuries, the development of addictive or obsessive behaviours and/or psychological distress [5, 6, 8, 9, 19, 20]. Some of the reasons attributed to the potential for harm include the use of non-evidence-based content and/or the use of content that is not designed to meet the specific health-related needs of individuals [5, 6, 8, 9, 20]. In this study, some of the social media influencers advertised and promoted themselves as qualified professionals, yet the content they shared was targeted at a generic audience and was not tailored to the specific needs of individuals. Furthermore, the content recommended by peers and family members was not always evidence-based. Hence, while the participants in this study were observed to be critical users of social media, engagement with health-related social media is highly complex and influenced by numerous personal and social factors, signalling the need for wider guidance for those who use social media, as well as those who recommend and endorse social media content.

Looking across the data, it can be argued that social media is an important and valuable inclusion to public health strategies to reach and engage mass audiences to support healthy lifestyle behaviours. The findings provide new evidence on key contextual factors that influence the use of social media for health, and include: the accessibility of the medium, work-home lifestyles, the capacity to foster community interaction, the development of authentic experiences, and whether content is recommended by individuals and/or organisations that are ‘trusted’. Based on these findings, we recommend that future public health strategies and social media interventions should carefully consider the contextual factors (work, home and lifestyle arrangements, pre-existing health-related knowledge and behaviours, and the perceived value of social media for health) that drive social media use for health behaviour change for diverse groups. In particular, evidence generated from this study suggests that social media strategies and interventions have the potential to effectively support behaviour change by prioritising social or community based interactions, creating authentic experiences of health behaviour change, and by ensuring that social media content is verified and/or accredited as being evidence-based.

The data reported demonstrated that physical activity levels prior to the lockdown and self-isolation status altered how social media was used in relation to health. Groups of people who are regularly physically active, value the community and social dimensions of social media use. Groups of people with low physical activity levels, value access to health-related information that focuses on the dualistic relationship between health and wellbeing. Older adults and/or people with medical conditions are competent users of social media, but tended to be more wary of privacy and misinformation and often require information specific to their health needs and body capabilities. These findings extend previous research that has tended to focus on the tailoring of social media content and health-related campaigns to specific health behaviours and/or the needs of different genders [46–48]. Our new evidence suggest that qualified professionals and practitioners could reach specific populations and groups (e.g. individuals with health conditions) through creating and sharing exercise and diet content and sessions on social media that are tailored to specific health needs and interests. In turn, we recommend that future public health strategies should implement and evaluate social media interventions that are tailored to the health-related needs of the target population, and that the design and format of social media content should be considered based on the interests of the target group, such as the choice of medium (Facebook vs WhatsApp), format (image, video, text) and space (private vs public).

This mixed methods study has provided a rich in-depth account from diverse perspectives on how social media use informs physical activity and diet-related behaviours, and perceptions of QoL, and the contextual factors that drive social media use for health-related behaviour change for diverse groups. The study has also some limitations mainly in relation to the characteristics of the sample and the ability to generalise the findings. The sample was mainly White British females aged 45 years, residing in areas of moderate levels of deprivation in the UK with few participants over the age of 65. The online recruitment approach for the survey and the recruitment and sampling for FGs also had the potential to recruit participants already engaging substantially with social media, therefore their perspectives may not reflect those of novice users of social media. Although the focus groups included participants from different COVID categories (e.g. self-isolating, leaving home to work, or working or studying from home) and with diverse health behaviours and uses of social media, they did lack representation from groups of people from across age groups and diverse ethnicities. Future studies will need to carefully design a very diverse recruitment plan in order to attract a larger and more representative sample and focus on exploring a wide range of social media use experiences.

## Conclusions

This study provides evidence to inform the development of robust guidance for health organisations on how they can design interventions to reach and engage with specific population groups through social media. Notably, participants in this study demonstrated that they were critical users of social media who used social media to derive benefit for their health and wellbeing. However, engagement with health-related social media is highly complex and influenced by personal and contextual factors. In turn, there is a need for wider guidance for those who use social media, as well as those who recommend and endorse social media content. Moreover, future public health strategies and social media interventions should carefully consider the contextual factors (work, home and lifestyle arrangements, pre-existing health-related knowledge and behaviours, and the perceived value of social media for health influenced) that drive social media use for health behaviour change for diverse groups and implement and evaluate approaches tailored to the needs of the target population.

## Supplementary Information

**Additional file 1.**

**Additional file 2.**

**Additional file 3.**

## Data Availability

The datasets used and/or analysed during the current study are available from the corresponding author on reasonable request.

## Abbreviations

UK: United Kingdom
USA: United States of America
COVID-19: Coronavirus-19
QoL: Quality of Life
PA: Physical Activity
SI: Self-isolating
W: Leaving home to work
H: Working or studying from home
FG: Focus Groups
IMD: Index of Multiple Deprivation

## Footnotes

1 Index of Multiple Deprivation in the UK accessed from: https://www.gov.uk/government/statistics/english-indices-of-deprivation-2019
