# Supplementary material for: Social media use informing behaviours related to physical activity, diet and quality of life during COVID-19: a mixed methods study
Source: BMC Public Health. 2021 Jul 6;21:1333. doi: 10.1186/s12889-021-11398-0 (PMC8259772; doi:10.1186/s12889-021-11398-0)
Supplement: Supplementary file 1 — Additional file 1. [file 12889_2021_11398_MOESM1_ESM.pdf]

# Consent Form

**Online Survey Consent Form**

We need to obtain your consent in order for you to proceed with the survey, so please click 'Yes' to the statements below if you would like to consent and take part.

I confirm that I have read and understand the information sheet and have had the opportunity to ask questions. ✱ *Required*

- Yes

All questions have been answered to my satisfaction. ✱ *Required*

- Yes

I understand that my participation is voluntary and that I am free to withdraw at any time up to two weeks after submission of my data without giving any reason or my rights being affected. ✱ *Required*

- Yes

I give consent for the data that I provide to be used for research purposes. ✱ *Required*

- Yes

Are you under the age of 16? ✱ *Required*

- Yes
- No

# Consent Form

If you would like to receive a summary of the results please tick the relevant box below.

After the survey we will also be conducting interviews, please tick the box if you provide consent to be contacted after the survey. Participation in the interview is not compulsory.

If you have any more questions about the study, please feel free to contact us on the details on the information sheet.

I would like to receive a summary of the results of the study. ✱ *Required*

- ○ Yes
- ○ No

I would like to be contacted about further research (e.g. interview) ✱ *Required*

- ○ Yes
- ○ No

Email address *Optional*

## Demographic Information

**We are asking you these questions in order to generate your unique study ID number. This is so, if you choose to, we can withdraw your data at a later point.**

**For example, if your birthday is the 21st, you were born in 1955, have 3 siblings, and your house number is 16, your study number would be: 21-1955-3-16.**

What is the date you were born? (E.g., if you were born on the 7th of December, please write 7). ✱ *Required*

☐

What is the year you were born? ✱ *Required*

☐

How many siblings do you have? ✱ *Required*

☐

What is your house number (if applicable)? *Optional*

☐

# Demographic Information Part 2

This questionnaire ask questions about your background. Please let the researcher know if you have any questions. Your answers will be kept confidential and if you do not wish to answer certain questions, you do not have to.

What is your age? *Optional*

What is your date of birth? *Optional*

More info

Gender identity: Which of the following describes how you think of yourself?

- Male
- Female
- In another way

If you selected 'In another way', please enter further information in this box

What is your current country of residence? *Optional*

What is your ethnicity?

- White - British
- White - Irish
- Other white background
- White and Black Caribbean
- White and Black African

- White and Asian
- Indian
- Pakistani
- Bangladeshi
- Chinese
- African
- Caribbean
- Arab
- Other

If you selected Other, please specify:

What is your marital status?

- Single
- Married
- Separated
- Divorced
- Widowed

How many children do you have?

How many years have you lived in the UK?

What is your highest level of education?

- No qualifications
- Primary school
- Secondary school
- College

- University/Higher education

What is your faith/religion?

- No religion
- Buddhist
- Christian
- Hindu
- Jew
- Muslim
- Sikh
- Other

If you selected Other, please specify:

What is your postcode?

How many people are currently living in your household, including yourself?

Do you currently work for pay?

- Working part time
- Working full time
- No

Are you retired from work?

- Yes
- No

Have you ever worked in the UK?

- ○ Yes
- ○ No

## Social Media

In this section, we will ask you about your uses of social media. Social media here refers to interactive technologies where you can create and share text, images, videos – such as Facebook, Twitter, Instagram, LinkedIn, Pinterest SnapChat, TikTok, WhatsApp and YouTube.

On average how many hours do you use social media per day?

Please don't select more than 1 answer(s) per row.

| | Less than 1 | 1-2 | 3-4 | 4-5 | More than 5 |
|---|---|---|---|---|---|
| Prior to the lockdown period | ☐ | ☐ | ☐ | ☐ | ☐ |
| In the last week | ☐ | ☐ | ☐ | ☐ | ☐ |

What are the main social media platforms that you use (you can select multiple answers if you wish)?

- ☐ Facebook
- ☐ Twitter
- ☐ Instagram
- ☐ LinkedIn
- ☐ SnapChat
- ☐ TikTok
- ☐ WhatsApp
- ☐ YouTube
- ☐ Pinterest
- ☐ Other
- ☐ None

If you selected Other, please specify:

Overall, has your use of social media changed as a result of the restrictions due to the COVID-19 lockdown period?

| | Yes – it has decreased a lot | Yes – it has decreased a little | No - It has stayed the same | Yes - It has increased a little | Yes - It has increased a lot |
|---|---|---|---|---|---|
| Social media | ☐ | ☐ | ☐ | ☐ | ☐ |

## Your Health

How would you describe your health compared to other persons of your age?

- Excellent
- Very Good
- Good
- Fair
- Poor

Have you ever been diagnosed with any of these diseases?

- No
- Hypertension
- Diabetes
- Heart disease and stroke
- Hyperlipidaemia
- Renal disease
- Osteoporosis
- Arthritis
- Cancer
- Other disease

If you selected Other, please specify:

Do you currently take any medication?

- Yes
- No

If you selected 'Yes', please give details of the medication.

Do you currently smoke?

- Yes
- No

If you selected 'Yes', please give details of the number of cigarettes per week.

Did you use to smoke?

- Yes
- No

If you selected 'Yes', please give details of how many years you smoked cigarettes.

Do you drink alcohol?

- Yes
- No

If you selected 'Yes', please give details of how many units of alcohol you drink on average in one week.

More info

What is your current living situation?

- I am currently self-isolating due to medical advice
- I am currently self-isolating due to development of symptoms
- I am currently self-isolating due to having been in contact with people who have developed symptoms
- I am only leaving my house to go to work, to get medication, to do food shopping and my daily exercise
- I am only leaving my house to get medication, to do food shopping and my daily exercise
- I am leaving my house as usual.
- Other

If you selected Other, please specify:

To what extent are you concerned about the following situations:

| | Not at all concerned | Unconcerned | Neutral | Concerned | Very concerned |
|---|---|---|---|---|---|
| a. You could test positive for COVID-19 | ☐ | ☐ | ☐ | ☐ | ☐ |
| b. A member of your family could test positive for COVID-19 | ☐ | ☐ | ☐ | ☐ | ☐ |
| c. You are less able to be physically active because of the restrictions due to COVID-19 | ☐ | ☐ | ☐ | ☐ | ☐ |
| d. You are sitting more because of the restrictions due to COVID-19 | ☐ | ☐ | ☐ | ☐ | ☐ |
| e. You are not able to see family and/or friends because of the restrictions due to COVID-19 | ☐ | ☐ | ☐ | ☐ | ☐ |
| f. You are not able to have access to sufficient food | ☐ | ☐ | ☐ | ☐ | ☐ |
| g. You are not able to have sufficient quantity of healthy food | ☐ | ☐ | ☐ | ☐ | ☐ |

# Nutrition/Diet

Please complete the following five questions related to how your consumption of food, beverages, and nutritional supplements may have changed during the lockdown period resulting from the Covid-19 pandemic.

| | Strongly disagree | Disagree | Neutral | Agree | Strongly agree |
|---|---|---|---|---|---|
| The types and amounts of food I consume have changed since the COVID-19 lockdown period started. | ☐ | ☐ | ☐ | ☐ | ☐ |

If you answered 'agree' or 'strongly agree' to the question above, please list the most significant changes (up to 3) in the types/amount of food you consume.

Non-alcoholic beverages

| | Strongly disagree | Disagree | Neutral | Agree | Strongly agree |
|---|---|---|---|---|---|
| The types and amounts of non-alcoholic beverages I consume have changed since the COVID-19 lockdown period started. | ☐ | ☐ | ☐ | ☐ | ☐ |

If you answered 'agree' or 'strongly agree' to the question above, please list the most significant changes (up to 3) in the types/amount of non-alcoholic berverages you consume.

Alcoholic beverages

| | Strongly disagree | Disagree | Neutral | Agree | Strongly agree |
|---|---|---|---|---|---|
| The types and amounts of alcoholic beverages I consume have changed since the COVID-19 lockdown period started. | ☐ | ☐ | ☐ | ☐ | ☐ |

If you answered 'agree' or 'strongly agree' to the question above, please list the most significant changes (up to 3) in the types/amount of alcoholic berverages you consume.

Nutritional supplements

| | Strongly disagree | Disagree | Neutral | Agree | Strongly agree |
|---|---|---|---|---|---|
| The types and amounts of vitamins, minerals and nutritional supplements I consume have changed since the COVID-19 lockdown period started. | ☐ | ☐ | ☐ | ☐ | ☐ |

List the most significant changes (up to 3) in the types/amount of vitamins, minerals and nutritional supplements you consume.

Eating behaviours

| | Yes – it has decreased a lot | Yes – it has decreased a little | No - it has stayed the same | Yes - it has increased a little | Yes - it has increased a lot |
|---|---|---|---|---|---|
| Overall, has the quality of your diet and nutrition changed as a result of the restrictions due to the COVID-19 lockdown period? | ☐ | ☐ | ☐ | ☐ | ☐ |

List up to 3 positive changes in the quality of your diet and eating behaviours that have occurred.

List up to 3 negative changes in the quality of your diet and eating behaviours that have occurred.

Have you seen, read or watched anything on social media related to diet/nutrition (such as recipes, supplements, alcohol, smoothies, nutrient information) during the COVID-19 lockdown period?

- Yes
- No

Who was that information from? (you can select multiple answers if you wish)

Please select at least 1 answer(s).

- Government (or equivalent)
- Official Health Organizations (e.g. NHS, Sport England)
- News or TV Accounts (e.g. Sky News, BBC)
- Celebrities/Influencers
- Sports Performers/Athletes
- Friends
- Family Members
- Someone of similar age/interest to you
- Research
- Other

If you selected Other, please specify:

How was this information shared? *Optional*

- Video
- GIF
- Image
- Meme
- Text
- Story
- Live
- Other

If you selected Other, please specify:

What was this information about? *Optional*

- ☐ Recipes (e.g. for baking, dinners, lunches)
- ☐ Guidelines (e.g. recommended type or nutrients)
- ☐ Benefits of particular diets (e.g. immunity, skin)
- ☐ Weight Loss
- ☐ Things to do with family members (e.g. children, parents, brothers, sisters)
- ☐ Other

If you selected Other, please specify:

Did the social media post... (you can select multiple answers if you wish) *Optional*

- ☐ Change your attitudes about your diet
- ☐ Provide you with new understandings about diet
- ☐ Influence you to act on the information in the post, e.g. try a new recipe
- ☐ Prompt you to like or comment on the post
- ☐ Prompt you to re-post or share the post on social media with your followers
- ☐ Other

If you selected Other, please specify:

# Physical Activity

Choose one activity category that best describes your usual pattern of daily physical activities including activities related to house and family care, transportation, occupation, exercise and wellness, and leisure or recreational purposes.

More info

| | Level 1: Inactive or little activity | Level 2: Regularly with low levels of exertion | Level 3: Participate in aerobic or strength exercises for 20 - 60 minutes per week | Level 4: Participate in aerobic or strength exercises for 1 - 3 hours per week | Level 5: Participate in aerobic or strength exercises for over three hours per week |
|---|---|---|---|---|---|
| Before COVID-19 | ☐ | ☐ | ☐ | ☐ | ☐ |
| During lockdown | ☐ | ☐ | ☐ | ☐ | ☐ |

## Continued...

During the last 7 days, on how many days did you do **moderate to vigorous** physical activities that took hard physical effort, and caused an increase in your heart rate and breathing? Examples are things like heavy lifting, digging in the garden, running, aerobics/exercise classes, and cycling? **Do not include walking.**

- None
- 1
- 2
- 3
- 4
- 5
- 6
- 7

How much time in total did you usually spend on one of those days doing moderate to vigorous physical activities? Please answer in hours and minutes.

During the last 7 days, on how many days did you **walk outside the house**? This includes walking at work, walking to travel from place to place, and any other walking that you did solely for recreation, sport, exercise or leisure.

- None
- 1
- 2
- 3
- 4
- 5
- 6
- 7

How much time in total did you usually spend walking on one of those days (please answer in hours and minutes)?

Overall, has the time you have spent doing physical activity and sitting changed as a result of the restrictions due to COVID-19 lockdown period?

| | Yes – it has decreased a lot | Yes – it has decreased a little | No - it has stayed the same | Yes - it has increased a little | Yes - it has increased a lot |
|---|---|---|---|---|---|
| Physical activity | ☐ | ☐ | ☐ | ☐ | ☐ |
| Sitting | ☐ | ☐ | ☐ | ☐ | ☐ |

How long have you spent sitting **per day** during the COVID-19 lockdown period?

Please don't select more than 1 answer(s) per row.

| | None | Less than 1 hour | 1 hour - 2 hours | 2 - 3 hours | 3 - 4 hours | 4 - 5 hours | 5 - 6 hours | 6 - 7 hours | More than 7 hours |
|---|---|---|---|---|---|---|---|---|---|
| a) | ☐ | ☐ | ☐ | ☐ | ☐ | ☐ | ☐ | ☐ | ☐ |

Have the types of physical activities you do changed as a result of the restrictions of COVID-19 lockdown period?

- ○ Yes
- ○ No

If yes, please describe how you have changed the type of physical activity you are now doing due to the COVID-19 lockdown.

Have you seen, read or watched anything on social media related to physical activity (such as exercise, workouts, information on walks, types of physical activity, or benefits) during the COVID-19 lockdown period?

- ☐ Yes
- ☐ No

Who was that information from? (you can select multiple answers if you wish)

Please select at least 1 answer(s).

- ☐ Government (or equivalent)
- ☐ Official Health Organizations (e.g. NHS, Sport England)
- ☐ News or TV Accounts (e.g. Sky News, BBC)
- ☐ Celebrities/Influencers
- ☐ Sports Performers/Athletes
- ☐ Friends
- ☐ Family Members
- ☐ Someone of similar age/interest to you

- [ ] Research
- [ ] Other

If you selected Other, please specify:

How was this information shared? *Optional*

- [ ] Video
- [ ] GIF
- [ ] Image
- [ ] Meme
- [ ] Text
- [ ] Story
- [ ] Live
- [ ] Other

If you selected Other, please specify:

What was the information about? *Optional*

- [ ] Workout (e.g. HIIT, yoga, stretch, resistance)
- [ ] Guidelines (e.g. recommended type or frequency of exercise)
- [ ] Benefits of physical activity (e.g. mental health)
- [ ] Body Image changes
- [ ] Things to do with family members (e.g. children, parents, brothers, sisters)
- [ ] Other

If you selected Other, please specify:

Did the social media post...(you can select multiple answers if you wish) *Optional*

Please select at least 1 answer(s).

- [ ] Change your attitudes about physical activity
- [ ] Provide you with new understandings about physical activity
- [ ] Influence you to act on the information in the post, e.g. try a new workout

- [ ] Prompt you to like or comment on the post
- [ ] Prompt you to re-post or share the post on social media with your followers
- [ ] Other

If you selected Other, please specify:

# Pain

We are interested in finding out about your experience of physical pain during the COVID-19 lockdown period. This is because we think the lockdown period might impact how our bodies work and feel.

Please rate your average levels of pain based on how you have felt during the lockdown period.

| | 1 (no pain) | 2 | 3 | 4 | 5 | 6 | 7 | 8 | 9 | 10 (worst imaginable pain) |
|---|---|---|---|---|---|---|---|---|---|---|
| a) | ☐ | ☐ | ☐ | ☐ | ☐ | ☐ | ☐ | ☐ | ☐ | ☐ |

During the lockdown period, how much has pain interfered with your work and daily activities? (including both work outside the home and housework)

Please don't select more than 1 answer(s) per row.

Please select at least 1 answer(s).

| | 1 (no pain) | 2 | 3 | 4 | 5 | 6 | 7 | 8 | 9 | 10 (worst imaginable pain) |
|---|---|---|---|---|---|---|---|---|---|---|
| a) | ☐ | ☐ | ☐ | ☐ | ☐ | ☐ | ☐ | ☐ | ☐ | ☐ |

Overall, has the amount of pain you experience changed as a result of the restrictions due to the COVID-19 lockdown period?

| | Yes – it has decreased a lot | Yes – it has decreased a little | No - it has stayed the same | Yes - it has increased a little | Yes - it has increased a lot |
|---|---|---|---|---|---|
| Pain | ☐ | ☐ | ☐ | ☐ | ☐ |

If you answered 'yes' to the question above, is this because you have a medical diagnosis which could be contributing towards, or causing the pain you are experiencing?

- ○ Yes
- ○ No

Have you seen, read or watched anything on social media related to pain during the COVID-19 lockdown period ?

- ☐ Yes
- ☐ No

Who was that information from? (you can select multiple answers if you wish)

Please select at least 1 answer(s).

- [ ] Government (or equivalent)
- [ ] Official Health Organizations (e.g. NHS, Sport England)
- [ ] News or TV Accounts (e.g. Sky News, BBC)
- [ ] Celebrities/Influencers
- [ ] Sports Performers/Athletes
- [ ] Friends
- [ ] Family Members
- [ ] Someone of similar age/interest to you
- [ ] Research
- [ ] Other

If you selected Other, please specify:

How was this information shared? *Optional*

- [ ] Video
- [ ] GIF
- [ ] Image
- [ ] Meme
- [ ] Text
- [ ] Story
- [ ] Live
- [ ] Other

If you selected Other, please specify:

What was the information about? *Optional*

- [ ] Causes/explanations for pain
- [ ] Exercises to reduce pain
- [ ] Medication to reduce pain
- [ ] Physiotherapy
- [ ] Other

If you selected Other, please specify:

Did the social media post...(you can select multiple answers if you wish) *Optional*

Please select at least 1 answer(s).

- ☐ Change your attitudes about pain
- ☐ Provide you with new understandings about pain
- ☐ Influence you to act on the information in the post, e.g. try a medication, exercise or contact a physiotherapist
- ☐ Prompt you to like or comment on the post
- ☐ Prompt you to re-post or share the post on social media with your followers
- ☐ Other

If you selected Other, please specify:

# Quality of Life

We are interested in finding out about how you perceive your quality of life and whether this has changed during the COVID-19 lockdown period.

Thinking about the lockdown period...

| | Very poor | Poor | Neither good nor poor | Good | Very good |
|---|---|---|---|---|---|
| How would you rate your quality of life? | ☐ | ☐ | ☐ | ☐ | ☐ |

Thinking about the lockdown period...

| | Very dissatisfied | Dissatisfied | Neither satisfied nor dissatisfied | Satisfied | Very satisfied |
|---|---|---|---|---|---|
| How satisfied are you with your health? | ☐ | ☐ | ☐ | ☐ | ☐ |

Overall, has your quality of life changed as a result of the restrictions due to the COVID-19 lockdown period?

| | Yes – it has decreased a lot | Yes – it has decreased a little | No - it has stayed the same | Yes - it has increased a little | Yes - it has increased a lot |
|---|---|---|---|---|---|
| Quality of life | ☐ | ☐ | ☐ | ☐ | ☐ |

List up to 3 positive changes in your quality of life that have occurred.

List up to 3 negative changes in your quality of life that have occurred.

Have you seen, read or watched anything on social media related to your quality of life during the COVID-19 lockdown period?

- [ ] Yes
- [ ] No

Who was that information from? (you can select multiple answers if you wish)

Please select at least 1 answer(s).

- [ ] Government (or equivalent)
- [ ] Official Health Organizations (e.g. NHS, Sport England)
- [ ] News or TV Accounts (e.g. Sky News, BBC)
- [ ] Celebrities/Influencers
- [ ] Sports Performers/Athletes
- [ ] Friends
- [ ] Family Members
- [ ] Someone of similar age/interest to you
- [ ] Research
- [ ] Other

If you selected Other, please specify:

How was this information shared? *Optional*

- [ ] Video
- [ ] GIF
- [ ] Image
- [ ] Meme
- [ ] Text
- [ ] Story
- [ ] Live
- [ ] Other

If you selected Other, please specify:

What was the information about? *Optional*

- [ ] Health
- [ ] Comfort
- [ ] Happiness
- [ ] Living conditions

- [ ] Other

If you selected Other, please specify:

Did the social media post...(you can select multiple answers if you wish) *Optional*

Please select at least 1 answer(s).

- [ ] Change your attitudes about your life
- [ ] Provide you with new understandings about your life
- [ ] Influence you to act on the information in the post
- [ ] Prompt you to like or comment on the post
- [ ] Prompt you to re-post or share the post on social media with your followers
- [ ] Other

If you selected Other, please specify:

# Social Media

In this section, we will ask you about your uses of social media. Social media here refers to interactive technologies where you can create and share text, images, videos – such as Facebook, Twitter, Instagram, LinkedIn, SnapChat, TikTok, Pinterest, WhatsApp and YouTube.

To what extent do you agree with the statement below?

Please don't select more than 1 answer(s) per row.

| | Strongly disagree | Disagree | Neutral | Agree | Strongly agree |
|---|---|---|---|---|---|
| Social media has been a good source of information on physical activity and diet/nutrition during the COVID-19.... | ☐ | ☐ | ☐ | ☐ | ☐ |

Overall, do you think social media during the COVID-19 lockdown period has had a positive influence on your engagement with physical activity, diet/nutritional behaviours, pain and/or quality of life?

Please don't select more than 1 answer(s) per row.

| | Strongly disagree | Disagree | Neutral | Agree | Strongly agree |
|---|---|---|---|---|---|
| Physical activity | ☐ | ☐ | ☐ | ☐ | ☐ |
| Diet/nutrition | ☐ | ☐ | ☐ | ☐ | ☐ |
| Pain | ☐ | ☐ | ☐ | ☐ | ☐ |
| Quality of life | ☐ | ☐ | ☐ | ☐ | ☐ |

Can you give an example of a social media post that has been particularly influential in your attitudes, knowledge and behaviors, and why? (Please feel free to insert a link to a particular social media post that is public access)
