# Supplementary material for: Social media use informing behaviours related to physical activity, diet and quality of life during COVID-19: a mixed methods study
Source: BMC Public Health. 2021 Jul 6;21:1333. doi: 10.1186/s12889-021-11398-0 (PMC8259772; doi:10.1186/s12889-021-11398-0)
Supplement: Supplementary file 2 — Additional file 2. [file 12889_2021_11398_MOESM2_ESM.docx]

**Supplementary File B**

**Table S1: Participant Characteristics and social media use for the whole sample**

|  |  | N | Country |  | Age | Gender |  | Ethnicity |  |  | IMD (UK only) | COVID context |  |  |
| --- | --- | --- | --- | --- | --- | --- | --- | --- | --- | --- | --- | --- | --- | --- |
|  |  |  | UK | Other |  | F | M | WB | OW | O |  | SI | W | H |
|  |  | 786 | 597 (76) | 98 (13) | 45.1 (19.4)  16-88 | 531  (69) | 235 (31 | 592 (77) | 104 (13) | 75  (10) | 6.3 (2.6) | 62 (8) | 190 (24) | 527 (67) |
| **Time on Social Media** | |  |  |  |  |  |  |  |  |  |  |  |  |  |
|  | Decrease | 62 (8) | 40 (7) | 9 (9) | 37.0 (17.0) | 48 (9) | 14 (6) | 39 (7) | 15 (14) | 8 (11) | 5.3 (2.8) | 4 (7) | 12 (6) | 45 (9) |
|  | No Change | 157 (20) | 119 (20) | 24 (25) | 501 (19.1) | 101 (19) | 55 (23) | 114 (19) | 23 (22) | 15 (20) | 64 (2.4) | 5 (8) | 34 (18) | 117 (22) |
|  | Increase | 563 (72) | 434 (73) | 66 (33) | 44.5 (19.4) | 380 (72) | 164 (70) | 435 (74) | 66 (64) | 52 (69) | 6.4 (2.6) | 53 (86) | 141 (74) | 364 (69) |
|  | Missing | 4 (1) | 4 (1) |  |  | 2 (0.4) | 2 (1) | 4 (1) |  |  |  |  | 3 (2) | 1 (0.2) |
| **During Lockdown** | |  |  |  |  |  |  |  |  |  |  |  |  |  |
|  | <1 hour | 111 (14) | 87 (15) | 15 (15) | 59.7 (17.1) | 63 (12) | 43 (18) | 88 (15) | 15 (14) | 5 (7) | 6.4 (2.4) | 13 (21) | 16 (8) | 82 (16) |
|  | 1-2 hours | 233 (30) | 183 (31) | 24 (25) | 48.9 (18.9) | 163 (31) | 64 (27) | 184 (31) | 35 (34) | 8 (11) | 6.4 (2.6) | 20 (32) | 65 (34) | 148 (28) |
|  | 3-4 hours | 209 (27) | 165 (28) | 23 (24) | 41.2 (17.6) | 144 (27) | 63 (27) | 160 (27) | 26 (25) | 22 (29) | 6.4 (2.7) | 14 (23) | 55 (29) | 137 (26) |
|  | 4-5 hours | 105 (13) | 76 (13) | 14 (14) | 40.2 (18.3) | 68 (13) | 34 (15) | 73 (12) | 9 (9) | 21 (28) | 6.5 (2.4) | 7 (11) | 26 (14) | 70 (13) |
|  | ≥5 hours | 113 (14) | 79 (13) | 17 (17) | 34.5 (16.7) | 85 (16) | 27 (12) | 82 (14) | 14 (14) | 15 (20) | 5.9 (2.5) | 7 (11) | 22 (12) | 83 (16) |
|  | Missing | 15 (2) | 7 (1) | 5 (5) |  | 8 (2) | 4 (2) | 5 (1) | 5 (5) | 4 (5) |  | 1 (2) | 6 (3) | 7 (1) |
| **Social Media Site^a^** | |  |  |  |  |  |  |  |  |  |  |  |  |  |
|  | Facebook | 548 (70) | 417 (70) | 75 (77) | 42.8 (18.1) | 392 (74) | 144 (61) | 413 (70) | 84 (71) | 41 (55) | 6.4 (2.6) | 39 (62) | 143 (75) | 361 (69) |
|  | Twitter | 278 (35) | 209 (35) | 28 (29) | 38.5 (15.9) | 175 (33) | 97 (41) | 209 (35) | 36 (35) | 31 (41) | 6.6 (2.6) | 11 (18) | 71 (37) | 195 (37) |
|  | YouTube | 422 (54) | 308 (52) | 69 (70) | 43.3 (19.8) | 262 (49) | 151 (64) | 299 (51) | 63 (61) | 54 (72) | 6.4 (2.6) | 33 (53) | 102 (54) | 282 (54) |
|  | WhatsApp | 590 (75) | 462 (77) | 62 (63) | 44.4 (185) | 415 (78) | 161 (69) | 453 (77) | 71 (68) | 58 (77) | 6.3 (2.6) | 45 (73) | 138 (73) | 401 (76) |
|  | Instagram | 379 (48) | 269 (45) | 56 (57) | 33.1 (14.2) | 258 (49) | 115 (49) | 270 (46) | 50 (48) | 55 (73) | 6.5 (2.6) | 17 (27) | 106 (56) | 252 (48) |

Note: ^a^ – participants could select more than 1 option; Female (F), Male (M); White British (WB), Other White (OW), Other (O); Self Isolating (SI), Leaving home to work (W), Working or Studying from Home (H); Index of Multiple Deprivation (IMD); United Kingdom (UK); ^b^ - data presented as number (%).

**Table S2: Contextual Factors that drove social media use for health-related behaviour change**

| Reason | Description (bold) and Illustrative Quotes (italics) |
| --- | --- |
| Free Access/Easy To Engage | **Free content that was accessible across devices (Tablet, phone), platforms (e.g. Android, Apple), delivered in a video format, and were short in duration, and that** **required no equipment or minimal/inexpensive ingredients**  *“The fact that his recipes are in 15 minutes, his workouts are short workouts…So, I think the fact that [celebrity personal trainer], everything is for quite short, busy people, isn’t it, and short timeframes? I think that’s why it works….My mates who’ve got kids have loved the daily workouts that he was doing from that side of things. I think it’s that, really, in terms of condensing, because the biggest thing, I think, a lot of people say they haven’t got time for exercise, when really… Yeah, but it’s good because I think he breaks that barrier down because, if people are honest with themselves, people have got 15 minutes or 20 minutes. You’ve just got to… Maybe not every day, but you’ve definitely just got to find a time to do it”. (Male 25-34)*  *“YouTube, it's just free, you can just access it whenever you want, there's no cost to it. That's another thing as well, because I'm not going to the gym, and I've frozen my membership because you've got to save money where you can… So, I think that's a big thing about it is the flexibility of it, and the fact that there's no cost to it either.” (Female, 35-44)* |
| Work-Home-Health Balance | **Less time spent commuting/travelling to work, more leisure time and the ease of completing online workouts at home and with the family.**  *You haven’t got the whole commute, you haven’t got the rush of, “Oh, god, I’m so hungry. When I get in from work I want to cook dinner and go to bed”, you haven’t got that. Our whole routines have been flipped, so everybody’s got more time on their hands. Also, I think, people are maybe realising, “Oh, actually, I don’t have to go to the gym to work out” or “I don’t have to join a class to work out.”… we both do Joe Wicks at 9:00 every morning, and we’ll do it together (Female 16-24)*  *“Since we spend a lot of time at home, we want to cook differently from usual. Usually we are in a hurry, you get something from the freezer and let’s go. We have gone through a lot of recipes on YouTube and tried them out.” (Male, 45-54)* |
| Creating E-Local Communities | **Content shared/posted by exercise groups and/or local sport and exercise facilities and/or cafes/restaurants (e.g. local gym classes, nutrition clubs, scouts and guides, football clubs and cooking classes) provided a sense of community, a ritual/schedule, and enabled participants to maintain social connections.**  *“My gym (in [UK county]), obviously, closed down very early on. They very quickly got active on Facebook. I think not many days go by without them putting something on there, a reminder of what time different classes are on, or where you can find them on Facebook and that sort of thing…. they’ve been very good…*  *If you couldn’t find a class you wanted organised by your own gym, there’d be one organised by another branch somewhere around the country. They seem to have been quite good. They’re free and you don’t have to be a member.” (Female, 55-64)*  *“So, I'll go on my bike, and I'll listen to my Audible and I've done one of the exercises [local gym name) on Facebook because it's more of a community feel... I need people to cheer me on and work out with; I can't do it on my own.” (Female, 45-54)*  *“We’ve got a WhatsApp running group, and I’ve got a Facebook Messenger running group. We all kept putting I’ve done this, I’ve done that, not the same as running with your friends or doing the parkrun, but it helps” (Male, 45-54)* |
| Authentic Experiences | **Participants were engaged with physical activity and diet videos or live stories when the presenter showed their real lives and personalities, by being warm, calm, and laidback and by opening their doors to their homes (babies and dogs), and this promoted feelings of in this together.**  *“I think [celebrity personal trainer]at the start of lockdown, you had a really strong sense of we're in it together, kind of thing. The whole country seemed to really be on board with what's happening, the whole clapping for the NHS and supporting [celebrity], those kinds of things. And [celebrity personal trainer]was another part of that… I tried one [celebrity personal trainer]class… and I do intend to do more.”*  *(Female, 35-44)*  *“Has to be his personality… I really liked it when he’d injured his wrist, the fact he kept going…and I liked it when he involved his wife in it…I just admire him for that. I just think he’s a nice, warm, likeable person, really. Like a friend that you want to try and please.” (Female 65-74)* |
| Recommended | **Peer/family members, official organisation (e.g. NHS, Diabetes UK, Runners World) or university professionals recommendations or endorsements for specific content influenced participants to use and engage with content.**  *“There’s a specific guy who I use all the time who a friend put me onto, who’s very, very good. He lays it all out with science. I think the guy’s got a biology degree, and he lays it all out in a way that you can actually understand. Rather than just being like, “Oh, this is the movement you need to do,” he actually lays out why you need to do it.” (Male, 16-24)*  *“I’ll go on pages such as [national sport organisation], which is a magazine that I’ve read before. I’ll go on trusted sources, or things that I see as trusted sources…* *Then, for example, I read… I spent, probably, half an hour reading three or four articles that all came from Facebook, from [national sport organisation]Group, based on nutrition and recovery, because somebody else had liked the same article. Then it was that classic: read one article and then, before you know it, you’ve read four or five different articles.” (Make, 25-34)* |

**Table S3 – Focus group description and participant characteristics**

| Focus Group | Description | Characteristics |
| --- | --- | --- |
| No Changes in Diet Quality | Included individuals who reported no change in diet quality on the survey | n=7, *M*age 55.14 ± 7.42 years, females=3, males=4 |
| Decreases in Physical Activity Levels | Included individuals who reported a decrease in physical activity levels on the survey | n=7, Mage 43.43 ± 19.70 years, females=6, males=1 |
| Increases in Physical Activity Levels | Included individuals who reported an increase in physical activity levels on the survey | n=8, Mage 47.13 ± 23.63 years, females=3, males=5 |
| Low Physical Activity Levels Prior to the Lockdown Period* | Included individuals who had low physical activity levels prior to the lockdown; refers to individuals who do not meet the recommended levels of physical activity per week (i.e. less than 150 minutes of moderate intensity aerobic physical activity or less than 75 minutes of vigorous-intensity per week) | n=10, Mage = 47.7 ± 14.55 years, females =8, males = 2 |
| High Physical Activity Levels Prior to the Lockdown Period* | Included individuals who had high physical activity levels prior to the lockdown; refers to individuals who exceeded the recommended levels of physical activity (i.e. more than150 minutes of moderate intensity aerobic physical activity or more than 75 minutes of vigorous-intensity per week) and reported engaging with three hours or more of aerobic or vigorous activity per week. | n=10, Mage 55.3 ± 21.85 years, females=6, males=4 |
| Use of Social Media as a Positive Resource for Physical Activity, Diet and/or QoL** | Included individuals who reported that social media was a positive resource for physical activity, diet and/or QoL in the survey | n=6, Mage 53.83 ± 13.70 years, females=4, males=2 |
| Increases in Time Spent on Social Media During the Lockdown Period | Included individuals who reported that time spent on social media during the lockdown period increased | n=10, Mage 48.70 ±21.52, females=5, males=5 |
| Self-Isolating Individuals | Included individuals who reported that they were self-isolating on the survey (see Table 1). | n=11, Mage 67.5 ± 7.42 years, females =6, males =5 |

********The classification of high physical activity levels was informed by our stakeholder consultations and the World Health Organisation guidelines for physical activity; **QoL = Quality of Life*
