# Supplementary material for: Social media use informing behaviours related to physical activity, diet and quality of life during COVID-19: a mixed methods study
Source: BMC Public Health. 2021 Jul 6;21:1333. doi: 10.1186/s12889-021-11398-0 (PMC8259772; doi:10.1186/s12889-021-11398-0)
Supplement: Supplementary file 3 — Additional file 3. [file 12889_2021_11398_MOESM3_ESM.docx]

**Figure S1: Thematic Map: Adults who were self-isolating and their engagement with social media**

**Restricted Access to Physical Activity Spaces**

Golf, Swimming &/or care home activities (table tennis pickle ball)

**Level 1 Context**

**Restricted Access to Supermarkets**

Reliance on online deliveries from supermarkets

**Restricted Social Interaction Outside of House Holds**

Visiting friends and family in-person prohibited

**Increase Online /Social Media Classes- Celebs**

Ballet, Yoga, Joe Wicks for Seniors, Zumba

**Level 2 Alternative Actions**

**Social Media to Maintain Social Contact**

Grandchildren, Family zoom quizzes

**Social Media to Maintain Social Hobbies**

Crafts, Gardening and Walking Groups, Book/Film Clubs & Church

**Adapt Diet**

Due to limited access to fresh fruit, vegetables and fish

**Local/Home Grown**

Increased home grown food in relation to gardening

**Increase Early Morning Leisure Activities**

Walking, Gardening, Cycling

**Level 3 Factors Influencing Actions (or Outcomes)**

**Word of Mouth/Publicity**

**Relevant to Senior Bodies**

**Knowledge/Experience**

Home cooking, gardening, balanced diet

**Trust/Privacy/Simplicity of Use**

Determined whether social media was used and/or specific sites

**Figure S2: Thematic Map: Adults who had high levels of physical activity prior to the lockdown and their engagement with social media**

**Restricted Engagement in Local Community/Professional Sport/PA**

Restricted access to social aspect of sport/PA (e.g. spectator, teams); Restricted Access to Physical spaces – gyms, equipment, professionals

**Level 1 Context**

**Level 2 Alternative Actions**

**Social Media As a Substitute/Add-on to maintain health, performance and quality of life**

**Level 3 Factors Influencing Actions (or Outcomes)**

**WhatsApp Groups**

Around clubs/sport - more messages, sharing information, memes

**Apps & Challenges**

Social networking for running/cycling – through Strava and Challenges (Run5Give5)

**Zoom/Facebook Live Workouts**

Led by gym instructors previously engaged with

**Instagram –**

**Home Gym**

Increased purchase of equipment

**Professionals**

Personal Trainers or Ex Pro’s workouts/ skills practice In

**Figure S3: Thematic Map: Adults with low levels of physical activity prior to the lockdown and their engagement with social media**

**COVID-19**

Re-emphasised the importance of physical and mental health

**Level 1 Context**

**Recipes**

Social media recipes from Instagram & Pinterest

**Social Eating**

More snacking, treats and alcohol with family

**Family Exercise**

Increase biking, walks, gardening

**Online Workouts**

Celebrity Personal Trainers – perceived higher quality than local gyms

**Local Online Cooking Classes**

Local restaurants Facebook live

**Level 2 Alternative Actions**

**Work Lifestyle**

Working from home, less time spent on commuting or travel

**Home Lifestyle**

Increased time at home: Leisure time in the home and with family members

**Level 3 Factors Influencing Actions (or Outcomes)**
